# Zonisamide-Induced Hallucinations: An Anticonvulsant’s Psychosis

**DOI:** 10.7759/cureus.16400

**Published:** 2021-07-15

**Authors:** Abdul Rahman Al Armashi, Patil Balozian, Francisco J Somoza-Cano, Kanchi Patell, Keyvan Ravakhah

**Affiliations:** 1 Internal Medicine, St. Vincent Charity Medical Center, Cleveland, USA

**Keywords:** neurological complication, zonisamide, anticonvulsant, anti-epileptic drug, partial seizure, hallucinations, psychosis, altered mental status, encephalopathy, neurological manifestations

## Abstract

Zonisamide is a new-generation anticonvulsant that works by altering the sodium and T-type calcium channels in the brain. It is currently approved for partial seizures, and trials are ongoing to evaluate the effectiveness against mania and chronic pain in adults. Psychosis is a rare side effect with an incidence of 2%. Our patient, a 52-year-old female with a past medical history of osteoarthritis and chronic pain only relieved by zonisamide is brought to the emergency department (ED) after a two-day history of altered mental status, agitation and visual hallucinations. One month prior, she had undergone total knee arthroplasty complicated with right knee cellulitis managed by IV (intravenous) long-term antibiotics of vancomycin and ertapenem. Physical examination was remarkable for disorientation to person, place, and time with intact remainder of the neurological exam. Initial laboratory work was unremarkable and a computerized tomography (CT) scan of the brain showed no acute intracranial abnormalities. The patient was treated as ertapenem-induced with altered mental status and the antibiotic was switched to meropenem upon discharge. Two weeks later, the patient presented to the ED with similar non-resolving complaints. As the patient’s symptoms didn’t improve after ertapenem discontinuation, the decision was made to stop zonisamide and carefully monitor for possible withdrawal symptoms. Progressively, our patient had a timely resolution of symptoms with a full return to baseline within a week. This case demonstrates the potential severity of zonisamide-induced psychosis. Additional studies are warranted to analyze the mechanism explaining its neurological side effect profile.

## Introduction

Zonisamide is a new-generation anticonvulsant that works by altering the sodium and T-type calcium channels in the brain [1]. It is currently approved for partial seizures, and trials are ongoing to evaluate the effectiveness against mania and chronic pain in adults [2-4]. Psychosis is a rare side effect with an incidence of 2% [2]. We are presenting a 52-year-old female who was admitted for altered mental status and psychosis. After extensive workup, zonisamide was found to be the offending agent.

## Case presentation

**Figure 1:**
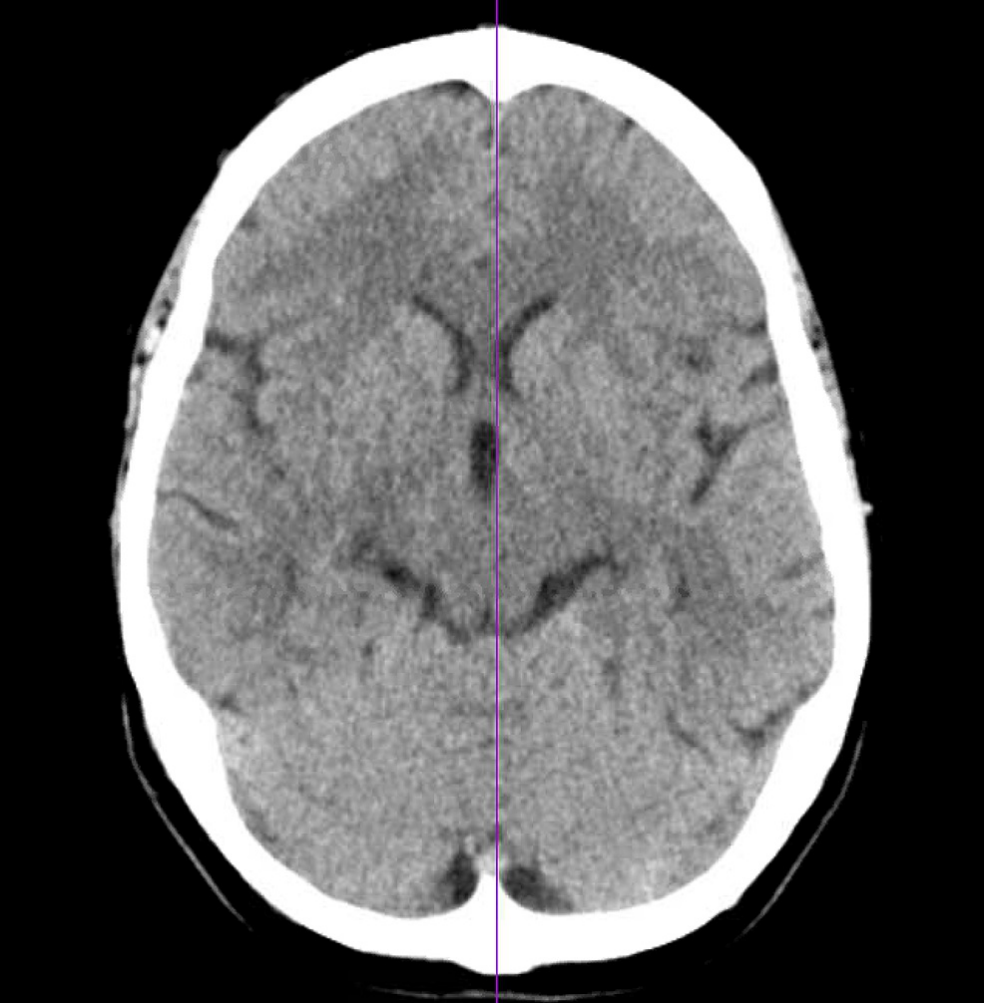
Computerized tomography scan of the brain. The computerized tomography (CT) scan of the brain showed no acute intracranial bleed, midline shift, or mass effect.

A 52-year-old female with a past medical history of osteoarthritis and chronic pain on zonisamide that was started four months prior to presentation as her osteoarthritis was refractory to multiple analgesia protocol regimens. She was brought to the emergency department (ED) after a two-day history of altered mental status, agitation and visual hallucinations. One month prior, she had undergone total knee arthroplasty complicated with right knee cellulitis. Cultures grew extended-spectrum beta-lactamase Escherichia coli, for which a peripherally inserted central catheter (PICC) was required for long-term antibiotics. On admission to the ED, one week had transpired since she had been receiving intravenous (IV) vancomycin and ertapenem. Physical examination was prominent for disorientation to person, place, and time with intact cranial nerves exam, normal upper and lower extremity power, with an unremarkable sensory and cerebellar exam. Her PICC line remained in situ. No signs of acute inflammation were noted at the insertion site. Initial laboratory work was unremarkable and a computerized tomography (CT) scan of the brain showed no acute intracranial abnormalities (Figure 1). The patient was treated as an ertapenem-induced altered mental status and the antibiotic was switched to meropenem upon discharge. Two weeks later, the patient presented to the ED with similar complaints as her symptoms deteriorated. Further work-up revealed once again an unremarkable metabolic panel, electrolytes and ammonia level within normal limits, non-revealing inflammatory markers, non-reactive syphilis, and a negative autoimmune panel. As the patient’s symptoms did not improve after ertapenem discontinuation, the decision was made to stop zonisamide. Progressively, our patient had a timely resolution of symptoms with a full return to baseline within a week.

## Discussion

Zonisamide is a novel anti-epileptic drug (AED) first described by Uno et al. in 1972 [5]. Currently, it is approved in the United States as an adjunctive treatment of partial seizures in adults [2]. Lately, it has been used for chronic pain as multiple trials have shown that zonisamide is effective in pain management and mania [3,4]. Furthermore, zonisamide is a 1,2-benzisoxazole-3-methanesulfonamidebenzisoxazole synthetic derivative that is metabolized through cytochrome P450 isozyme 3A4. Interestingly, it is the first compound from the sulfonamide group of chemicals to be used as an AED [6]. 

The anticonvulsant properties of zonisamide are multifaceted. It includes the inhibition of T-type calcium channels in neurons, leading to the suppression of neuronal hypersynchronization and decreasing the spread of seizure activity across the cell. Additionally, It affects the sodium channel, causing action potentials to fire at a lower frequency over longer periods of time. Zonisamide also inhibits carbonic anhydrase; however, this is not considered part of the drug's anti-epileptic properties [1]. 

Side effects of zonisamide are mainly of the GI system, including anorexia and abdominal pain. Neurological side effects are primarily dizziness and drowsiness. Psychosis has been rarely reported, with an incidence of 2% [2]. Additionally, the Naranjo approach is a proven method for the evaluation of a possible association between a clinical side effect and a drug. The Naranjo score for our patient was 6, indicating that the side effect was probable. A probable score implies that the reaction to the suspected drug followed a known temporal sequence and was confirmed by drug withdrawal. Finally, the event could not be explained by the known clinical status of the patient [7].

Upon literature review, four reported cases were found (Table 1), the median age was 37 (range 30-50), affecting both genders equally, and symptoms started between 1 and 10 months after starting zonisamide. Nevertheless, our patient was similar to the reported cases where symptoms completely resolved after medication discontinuation [8-11]. In Japan, a retrospective study was done by Miyamoto et al. on 74 epileptic patients on zonisamide treatment. Psychosis was observed in 14 patients; 12 of them were males. Notably, the emergence of psychotic symptoms varied between immediate onset to months and years after starting the zonisamide [12].

**Table 1:** Literature review.

Case	Age	Gender	Duration of zonisamide treatment	Symptoms	Outcome
Charles et al. 1990 [8]	31	Male	1 month	Auditory hallucinations and delusions of grandeur	Symptoms resolved after medication discontinuation
Michael et al. 2007 [9]	50	Female	6 months	Auditory and visual hallucinations	Symptoms resolved after medication discontinuation
Abdoh et al. 2014 [10]	34	Male	10 months	Paranoia	Symptoms resolved after medication discontinuation
Platt et al. 2014 [11]	34	Female	1 month	Psychosis	Symptoms resolved after medication discontinuation

## Conclusions

This case demonstrates the potential severity of zonisamide-induced psychosis. Zonisamide should be kept as a differential diagnosis of altered mental status and psychosis after excluding common medical causes. Additional studies are warranted to analyze the mechanism behind its neurological side effect profile as well as to assess the causation and correlation between this medication and altered mental status.
